# Electrical cortical stimulation can impair production of the alphabet without impairing counting

**DOI:** 10.1016/j.ebr.2021.100433

**Published:** 2021-02-11

**Authors:** Paulina Henriquez Rojas, Adithya Sivaraju, Imran H. Quraishi, Michael Vanderlind, Adrià Rofes, Monika M. Połczynska-Bletsos, Dennis D. Spencer, Lawrence J. Hirsch, Christopher F.A. Benjamin

**Affiliations:** a Comprehensive Epilepsy Center, Department of Neurology, Yale University School of Medicine, New Haven, CT, USA; b Department of Neurolinguistics and Language Development, University of Groningen, Groningen, the Netherlands; c Department of Psychiatry and Biobehavioral Sciences, David Geffen School of Medicine at UCLA, USA

**Keywords:** Electrocorticography, Language mapping, Letters, Numbers, Superior temporal gyrus

## Abstract

Neurosurgery has the potential to cure patients with drug-resistant focal epilepsy, but carries the risk of permanent language impairment when surgery involves the dominant hemisphere of the brain. This risk can be estimated and minimized using electrical stimulation mapping (ESM), which uses cognitive and linguistic tasks during cortical ESM to differentiate “eloquent” and “resectable” areas in the brain. One such task, counting, is often used to screen and characterize language during ESM in patients whose language abilities are limited. Here we report a patient with drug-resistant epilepsy arising from the language-dominant hemisphere using fMRI. Our patient experienced loss of the ability to recite or write the alphabet, but not to count, during ESM of the dominant left posterior superior temporal gyrus. This selective impairment extended to both spoken and written production. We suggest the need for caution when using counting as a sole means to screen language function and as a method of testing low functioning patients using ESM.

## Highlights

• Counting is used in Electrical Stimulation Mapping (ESM), often as a language screen.
• We report a case where ESM reliably disrupted production of letters, but not numbers.
• Counting is not an accurate screen of all language functions in ESM.

## Graphical abstract

**Figure:**
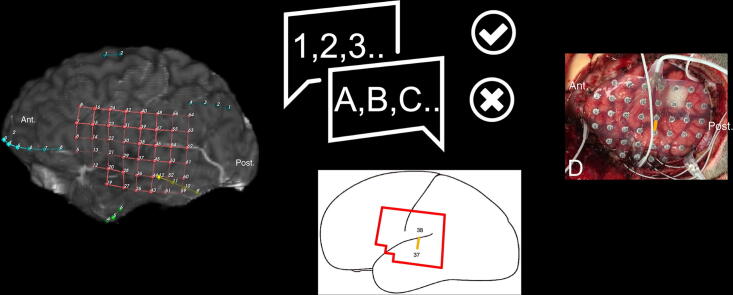

## 1 Introduction

Electrical stimulation mapping (ESM) is paired with cognitive testing to identify language cortex in neurosurgical planning. When language is consistently disrupted, the stimulated cortex is assumed to be language-critical and is therefore preserved during surgery. The tasks currently used across epilepsy surgical programs are not standardized: epilepsy centers use one (1%), two (18%), three (29%) or four (52%) measures of “production”, “comprehension”, “naming” and “reading” tasks to guide surgical margins [1]. One such task, counting aloud, is often used to screen language function [2], [3], or to map language in patients unable to perform more complex linguistic tasks [4].

Numbers and letters are cultural inventions that do not hold meaning for individuals who have not learned them, such as infants and illiterate adults [5], [6]. Both may be represented in multiple ways, including symbolically and non-symbolically [7], and as automated motor sequences [4]. However, numbers have multiple semantic properties that letters lack. These include numerical meaning, parity, multiplicativity, and magnitude [8], and suggest neural representations of numbers and letters might be at least partially separable.

Neuroimaging research on healthy controls is consistent with this view [9]. Recognition and processing of numerical symbols engages the dominant intraparietal sulcus to a greater extent than the processing of letters (e.g. [7]), with a recent meta-analysis indicating numerical processing involves the parietal cortex more broadly [10]. A region of inferior temporal cortex (right hemisphere moreso than left), the 'Number Form Area' (NFA), has been shown to be more engaged in identifying numbers than letters or other symbols (false fonts) [11]. This region is specialized for numeral identification even in congenitally blind individuals [11]. Of note, however, it remains possible that the less prominent left hemisphere Number Form Area is subsumed by the Visual Word Form Area (VWFA). Of note, both the NFA and VWFA are able to be engaged in blind individuals, indicating that these regions are not specific to *visual* symbols representing numbers and words [11]. Further, the processing of single letters and the processing of letter strings engage other regions residing within left fusiform cortex [12]. Arithmetic calculation and magnitude comparison, however, tend to engage parietal cortex more bilaterally [13], [14], [15], [16]. Moreover, magnitude processes in the horizontal intraparietal sulcus, elicited by alphabetic and numeric stimuli, seem to favor number recognition. Whereas, for letters an increased working memory load is needed to trigger these processes (ordinal sequence of the alphabet). These findings suggest simultaneous, different processes taking place for a specific function and that numbers are more easily accessed than letters, and thus their exclusive use for language assessment is debatable [17].

The potential for lesions to selectively impact aspects of letter and number processing has been reported in different studies. Anderson and colleagues [18], for instance, described a patient who suffered alexia and agraphia selectively impacting single words and letters, but not numbers. This deficit followed resection of a left posterior middle frontal gyrus (BA6) lesion. The patient remained able to orally spell words without issue, however. Starrfelt [19] reported a case with no clear lesion (MRI, CT, SPECT) following a concussion, who suffered alexia and agraphia for letters, but not numbers. He remained able to orally spell words. Conversely, in patients with dementia, alexia may affect numbers but not letters (e.g. [20]).

Here we provide further evidence of a dissociation between the production of letters and number during ESM. This finding was observed during stimulation of the dominant left posterior superior temporal gyrus (STG) in an adult undergoing planning for potential neurosurgical treatment of drug-resistant epilepsy.

## 2 Materials and methods

### 2.1 Case history

The patient was a 45-year-old, right-handed female with unremarkable developmental, neurological and psychiatric history. She spoke English primarily and a Baltic language secondarily. She completed graduate studies and had worked professionally. Seizures began at age 17. In her early 20 s a left anterior temporal cavernous angioma was identified and resected. Seizures recurred after a few years’ of post-operative seizure freedom. Her primary seizures were focal aware non-motor, involving an epigastric sensation, fear, expressive language dysfunction and inconsistent receptive aphasia. Seizure duration was 15–20 s and frequency was 10 seizures monthly at the time of evaluations. Infrequently, seizures progressed to involve greater language disturbance. Even less frequently, focal seizures progressed to become bilateral tonic-clonic seizures.

### 2.2 Investigations

**Fig. 1:**
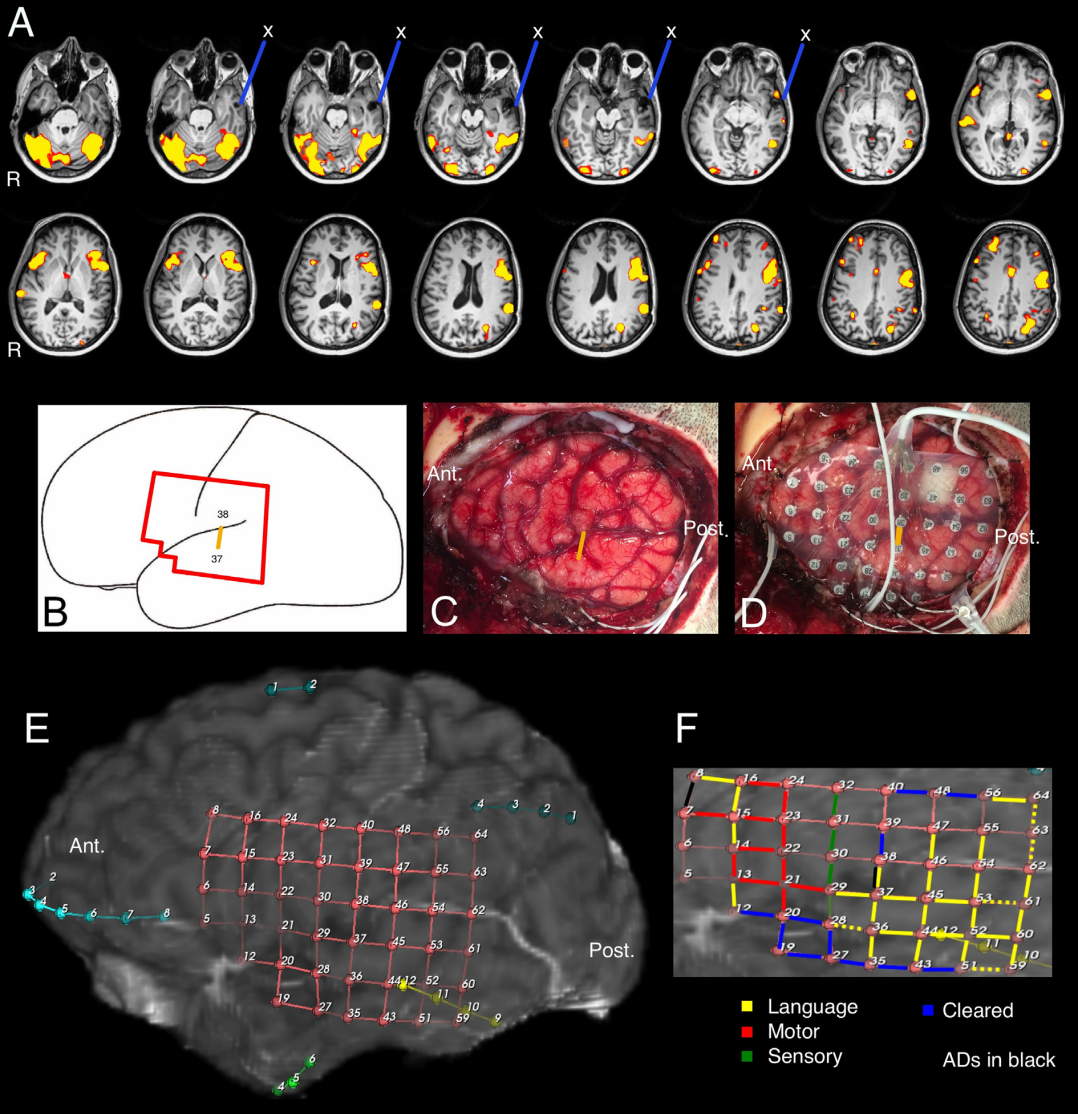
Results of language mapping (fMRI, ESM) and electrode placement. (A) MRI & language fMRI. The right of the images is the left side of the brain. Overlap was present for visual object naming; auditory responsive naming; and verbal responsive naming tasks. Historic resection (X) is shown with  lines. The location of contacts 37 & 38 (B) is shown schematically and (C, D) at reflect the surgical field and implant. (E) The estimated placement was approximated through three-dimensional registration of CT image to MRI. (F) The ESM results resulted in pairs of contacts connected by  lines that are language positive, lines that are  indicates motor,  lines were positive for sensory stimuli, and  sites were cleared a non-eloquent. Dotted lines indicate ESM association at low amperage.

Video-EEG showed left anterior temporal seizure onset and left regional temporal evolution. PET showed hypometabolism in the area of prior resection. MRI showed a 7 mm lesion (thrombosed pseudoaneurysm vs venous varix) in the medial aspect of the prior resection and the site of prior left superior and middle temporal gyral resection (Fig. 1A; blue line). Language fMRI was left hemisphere dominant with some bilateral representation in Wernicke's area (Fig. 1A). Neuropsychological assessment was Average (Full-scale IQ = 104) with impaired naming (Boston Naming Test II Z = −2.9), phonemic fluency (Z = −2.8) and comprehension (Boston Diagnostic Aphasia Examination Z = −1). The latter was judged to be artifactual. There was intact verbal memory.

#### 2.2.1 Electrical stimulation mapping protocol

Extraoperative electrical stimulation mapping was completed across three days. There was continuous electrocorticography (ECoG) recording for the entire duration of the patient stay, including during ESM. Stimulation was completed using a current-controlled Nicolet Cortical Stimulator (Natus Medical Incorporated) with 1–5 second trains of 50 Hertz, bipolar, biphasic, rectangular waveforms of 0.3 milliseconds pulse. Stimulation was first screened from 1 to 5 mA, in 1 mA steps, while the patient counted aloud (1–20) and (sporadically) recited the alphabet. Testing continued in 1 mA increments with the naming of visual objects until a stimulation endpoint was reached. Stimulation endpoints were observed for language disturbance; occurrence of afterdischarges (ADs), or a maximum of 12 mA stimulation was reached. At the highest stimulation level, six tasks were completed. (1) Counting from 1 to 20 was completed by the patient without specific stimuli. Tasks using visual stimuli included (2) naming visual objects (pictures); (3) following written commands; and (4) reading a paragraph aloud. Task with auditory stimuli included (5) naming auditorily described objects and (6) following auditory commands. While counting and object naming could have been tested using shorter train duration (often 1–2 s), though testing comprehension often required longer train duration (typically 3–5 s) to adequately test function, although the latter had a higher chance of producing ADs.

## 3 Results

On ESM, Broca’s and Wernicke’s areas were identified with extensive posterior language representation (Fig. 1F). The key finding occurred in the posterior superior temporal gyrus (contacts 37–38; Fig. 1B–D) during language screening. During stimulation the patient was able to count from 1 to 20 but unable to recite the alphabet on any of three trains of 3 stimulations (Table 1). This result was confirmed the following day when recitation of the alphabet was again halted on 3/3 stimulations on three separate occasions. However, counting was not disrupted on any of the three stimulation sessions, though there was hesitation in one instance.

**Table 1:** Electrical Stimulation Mapping Results. Numbers shown (Y/Z) represent accuracy during stimulation; the number of successful responses (Y) as a proportion of the stimuli given under stimulation (Z). All stimuli were auditory. Stimulation occurred for 1–2 seconds' duration, which was sufficient to disrupt function. These findings were observed starting at 5 Ma through 9 mA (see methods for further detail). ⧫These findings were obtained and reproduced across two separate days.

Further testing on day two showed selective disruption of letters, but not numbers, in written output. The ability to write the alphabet was stopped on each of eight stimulations. Again, when asked to write the numbers 1 to 20, production was not disrupted on any of eight stimulations.

When stimuli were not written in sequence (i.e., were written in response to dictation), similar results were obtained. The patient could only write letters during stimulation on five of twelve tasks (42% accuracy). Writing of random digits was typically unimpaired (80% accuracy; 10 trials).

These findings were observed starting at 5 mA and through 9 mA and using a train duration of 1–2 s, which was sufficient to disrupt counting. ECoG was carefully reviewed for any occurrence of ADs or seizures. Further language tasks could not be completed at the main contacts of interest in the posterior superior temporal gyrus (37–38). On day one, testing was halted due to recurrent ADs and seizures. On day two, testing of stimuli other than letters/numbers was halted by the patient (frustration) in addition to ADs. Testing comprehension at this contact pair was particularly challenging as it required longer train durations (typically 3–5 s) to present the stimuli, and this frequently invalidated the testing results due to persistent ADs and seizures.

Contact 37, on the superior temporal gyrus (STG), appeared central to these findings. With our standard tasks (above), naming, reading and repetition were disrupted at 5 mA across 37–45 (STG-STG), 37–29 (STG-STG) and 37–36 (STG-STG). Contacts 38–39, in the inferior anterior parietal cortex, were cleared (12 mA). Contacts 30–38 (STG-inferior parietal cortex) and 38–46 (inferior parietal cortex-STG) were not evaluated.

## 4 Discussion

To our knowledged, our case is the first to show a dissociation between the production of letters and numbers in both spoken and written output during ESM of the posterior STG. This finding suggests that during ESM, counting is not a reliable clinical screen for basic, overlearned language skills. This is important as the ability to count aloud is used by many epilepsy centers to gauge the voltage of the electrical current produced by the electrical stimulator, and as a simple speech task to map language when patients cannot complete more complex tasks (e.g. [8], [9]). They raise the possibility that in such cases the cortex may be inaccurately cleared and inadvertently resected. Indeed, 40% of epilepsy programs report instances where patients do suffer enduring language deficits when boundaries drawn using ESM are respected [1].

This case study is in line with prior lesional ESM studies as well as neuroimaging research on healthy controls that show a functional dissociation in the brain between numbers and letters (e.g. [9], [13], [18], [19]). The finding of this dissociation within a region of the posterior STG is novel. It also raises the possibility that this region could form a target for visual prosthetics in patients who have lost the ability to read, such as patients with temporo-occipital lesions including people with epilepsy.

We were limited in this case to ESM of these tasks at only one pair of contacts. This does not invalidate our findings. However, it does mean similar results could have been observed at other contact sites elsewhere. It is also possible these findings are impacted by current spread. This also does not detract from our result, though it may mean our findings require more diffuse cortical disruption within Wernicke’s area. A key concern in intracranial studies is the possibility of electrode movement. While the location of this patient's electrodes was confirmed, it is possible (though unlikely) that the electrodes could have shifted such that the superior contact abutted inferior parietal cortex.

This case suggests clinicians performing ESM should consider placing a low level of confidence in areas cleared using counting alone, and underscores the urgent need to develop an evidence-based, widely available protocol for ESM to investigate language function [1].

## Ethical statement

All authors are compliant with all relevant ethical regulations.

**Study funded** in part by American Academy of Neurology Clinical Research Training Scholarship – Benjamin, 2018–2020.

## Declaration of Competing Interest

The authors declare that they have no known competing financial interests or personal relationships that could have appeared to influence the work reported in this paper.
